# *Clonorchis sinensis* infection and co-infection with the hepatitis B virus are important factors associated with cholangiocarcinoma and hepatocellular carcinoma

**DOI:** 10.1007/s00436-017-5572-1

**Published:** 2017-08-12

**Authors:** Yunliang Shi, Zhihua Jiang, Yichao Yang, Peiqiu Zheng, Haiyan Wei, Yuan Lin, Guoli Lv, Qingli Yang

**Affiliations:** 1 Institute of Parasitic Disease Prevention and Control, Guangxi Zhuang Autonomous Region Center for Disease Control and Prevention, 18, Jinzhou Rd, Nanning, Guangxi 530028 China; 2 Hengxian People’s Hospital, Nanning, 530300 China; 3 Guangxi Key Laboratory for the Prevention and Control of Viral Hepatitis, Guangxi Zhuang Autonomous Region Center for Disease Control and Prevention, Nanning, 530028 China

**Keywords:** *Clonorchis sinensis*, Hepatitis B virus, Intrahepatic cholangiocarcinoma, Hepatocellular carcinoma

## Abstract

To evaluate the contributions of *Clonorchis sinensis* and hepatitis B virus to the development of cholangiocarcinoma (ICC) and hepatocellular carcinoma (HCC), *C*. *sinensis* and hepatitis B virus infections in 20 clinical liver cancer cases from a *C*. *sinensis*- and hepatitis B virus-epidemic region were detected. Eight cases of ICC, 11 cases of HCC and one mixed ICC and HCC case were verified by CT, pathological section and (or) observations during surgery. The *C*. *sinensis* infection was detected by stool microscopy and ELISA, and the worms and eggs found during surgery and in pathological sections also allowed for diagnoses. Hepatitis B virus infections were detected by ELISA. In the 20 cases, 18 patients were diagnosed with *C*. *sinensis* infections. Eight of the 20 patients were infected with the hepatitis B virus, and seven were co-infected with *C*. *sinensis*. In the eight ICC patients, seven were diagnosed with *C*. *sinensis* infection, and two had mixed infections with the hepatitis B virus. In the 11 HCC patients, 10 were diagnosed with *C*. *sinensis*, four had mixed infections with the hepatitis B virus, and only one HCC patient presented a single infection by the hepatitis B virus. These clinical observations revealed that *C*. *sinensis* infection and *C*. *sinensis* co-infection with the hepatitis B virus are important factors in ICC and HCC.

## Introduction

*Clonorchis sinensis* is a food-borne zoonotic parasite that is endemic predominantly in Asian countries such as China, Korea, Japan and Vietnam. Approximately 35 million people have been infected worldwide, and 15 million of these infected people were in China, according to a report based on a nationwide survey (Young et al. 2010; Qian et al. 2016). Previous epidemiological data showed that clonorchiasis is endemic in southeast China, especially in the Guangxi and Guangdong provinces (Chen et al. 2012). Hengxian County, located in Guangxi, is one epidemic region and has the highest known infection intensity. Some epidemiological evidence strongly implicates liver fluke infection in the aetiology of one of the liver cancer subtypes, cholangiocarcinoma (Hou 1956; Belamaric 1973; Papachristou et al. 2005; Choi et al. 2006; Lim et al. 2006), and the WHO-classified *C*. *sinensis* as a group I carcinogen-metazoan parasite that potentially induces cholangiocarcinoma in humans (Bouvard et al. 2009 **)**. However, large-scale clinical observations are still lacking. Whether *C*. *sinensis* infection contributes to hepatocellular carcinoma (HCC) remains inconclusive. Tan et al. showed that clonorchiasis is an important risk factor for HCC (Tan et al. 2008), but Shin et al. obtained conflicting results (Shin et al. 1996).

The hepatitis B virus (HBV) is more important in liver cancer, with 2 billion people infected worldwide, and more than 350 million are chronic carriers of the virus (Honer Zu Siederdissen and Cornberg 2014). Southeast Asia comprises countries, including China, where HBV is highly endemic, with approximately 170 million people who are chronically infected with HBV (Yang et al. 2014; Sun et al. 2002). Hepatitis B virus (HBV) infection can develop into liver diseases and cancers such as cirrhosis, hepatic decompensation and HCC (Rapti and Hadziyannis 2015). To evaluate the contribution of *C*. *sinensis* and the hepatitis B virus to the development of cholangiocarcinoma (ICC) and HCC, we observed 20 liver cancer patients from a hospital in Hengxian County, which has one of the highest *C*. *sinensis* infection rates in China.

## Materials and methods

From 2014 to 2015, a total of 20 liver cancer patients who visited the Hengxian People’s Hospital for treatment were observed for *C*. *sinensis* and hepatitis B virus infections, including 12 males and 8 females with an age range of 34–81 years. Progressive jaundice, upper abdominal pain, emaciation, fever, fatigue and inappetence were the main clinical manifestations of the patients. The patients underwent abdominal CT scans, and some patients underwent surgery as indicated by a doctor. Tumoural masses located in the bile duct and the liver were resected surgically. The removed pathological tissues were fixed in 4% formaldehyde, embedded in paraffin, sectioned, observed and HE stained to verify ICC and HCC.

Stool samples were collected from all patients and were examined by the formalin-ether sedimentation method to diagnose *C*. *sinensis*. The specific serum antibodies to *C*. *sinensis* were screened using an enzyme-linked immunosorbent assay (ELISA) kit (Kangbaidu, Shenzhen, China). The sera were diluted 1:100, and the absorbance was read at 490 nm using an ELISA reader (RT-6000, Rayto, Shenzhen, China). The worms and eggs found in biliary drainage fluids during surgery and pathological section were also used to diagnose *C*. *sinensis* infection. The HbsAg was detected using a kit from Abbott (Abbott Laboratories, North Chicago, IL, USA).

## Results

**Fig. 1:**
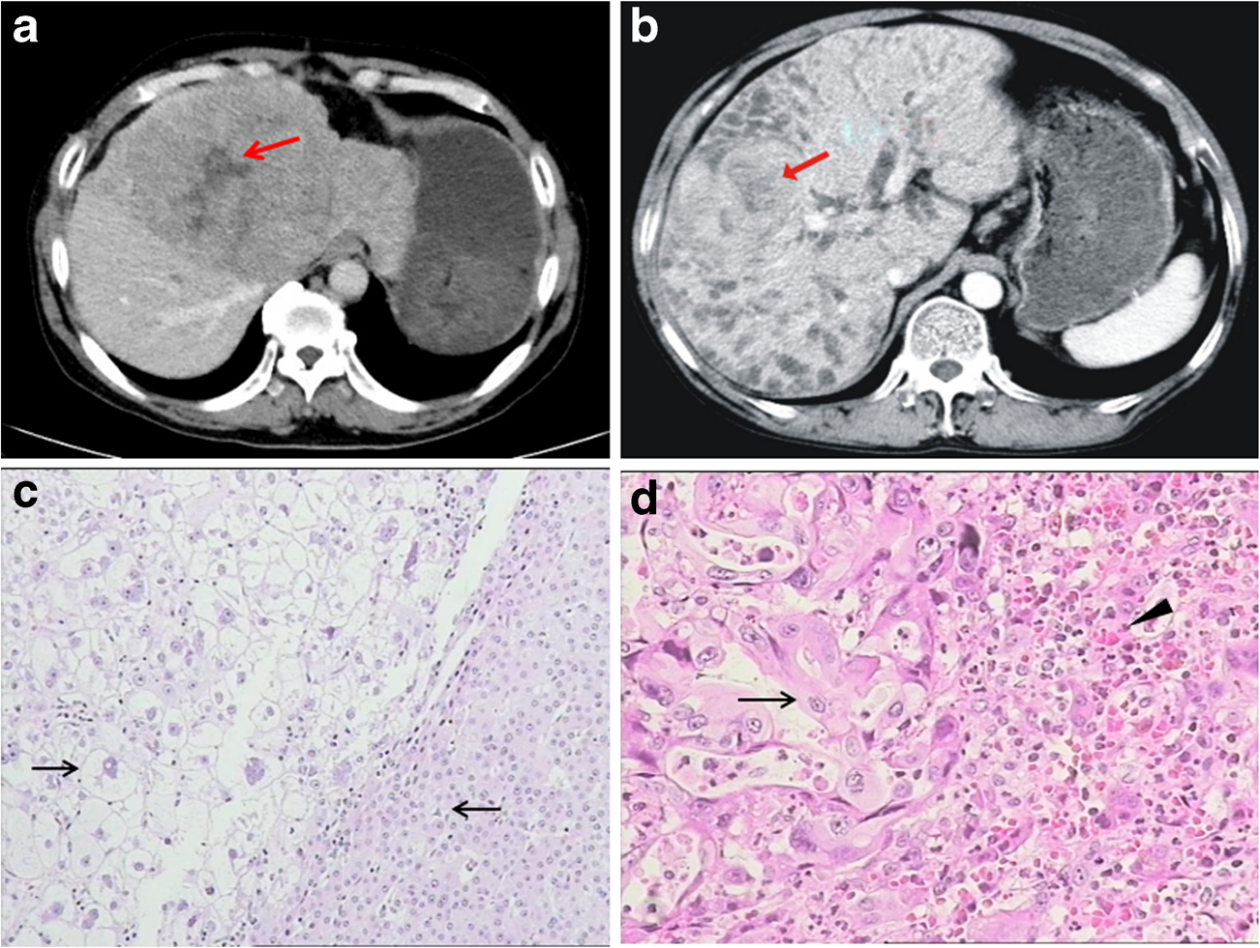
CT diagnosis and biopsy of cholangiocarcinoma and hepatocellular carcinoma. **a** CT diagnosis of hepatocellular carcinoma. **b** CT diagnosis of intrahepatic cholangiocarcinoma. **c** Liver biopsy of hepatocellular carcinoma (magnification ×400). **d** Liver biopsy of cholangiocarcinoma (magnification ×400)

In the 20 ICC and HCC patients, 8 had ICC, 11 had HCC and 1 had ICC with HCC (Table 1). Most of the ICC patients (5/8) infected with *C*. *sinensis* were not infected with HBV, two patients had mixed infections with HBV, and neither infection could be detected in one patient. In the 11 HCC cases, 6 patients were infected with only *C*. *sinensis*, 1 patient was infected with HBV, and 4 patients had a *C*. *sinensis* and HBV mixed infection. The abdominal CT scan showed an isodense lesion causing biliary obstruction in all patients. Intrahepatic ducts adjacent to the mass were dilated (Fig. 1b). Microscopically, the carcinomas had various sizes and were irregularly shaped with an adenoid-like structure, and the cancer cells showed atypia with an irregular nucleus and a prominent nucleolus. Around the cancer nests, fibre-reactive chronic inflammatory cells and eosinophils were apparent (Fig. 1c). In HCC, the carcinomas showed cord and nest structures, and some of the cancer cells had a transparent cytoplasm. In the nest-like thin-walled vessels around the cancer tissue, non-fibroblast responses and inflammatory cell infiltration were observed (Fig. 1d).

**Table 1:** The *C*. *sinensis* and hepatitis B virus infection in the 20 cases of ICC and HCC

Group	HCC	ICC	HCC + ICC	Total
*C*. *sinensis*	6	5	0	11
HbsAg+	1	0	0	1
*C*. *sinensis* + HbsAg+	4	2	1	7
Uninfected	0	1	0	1
Total	11	8	1	20

**Fig. 2:**
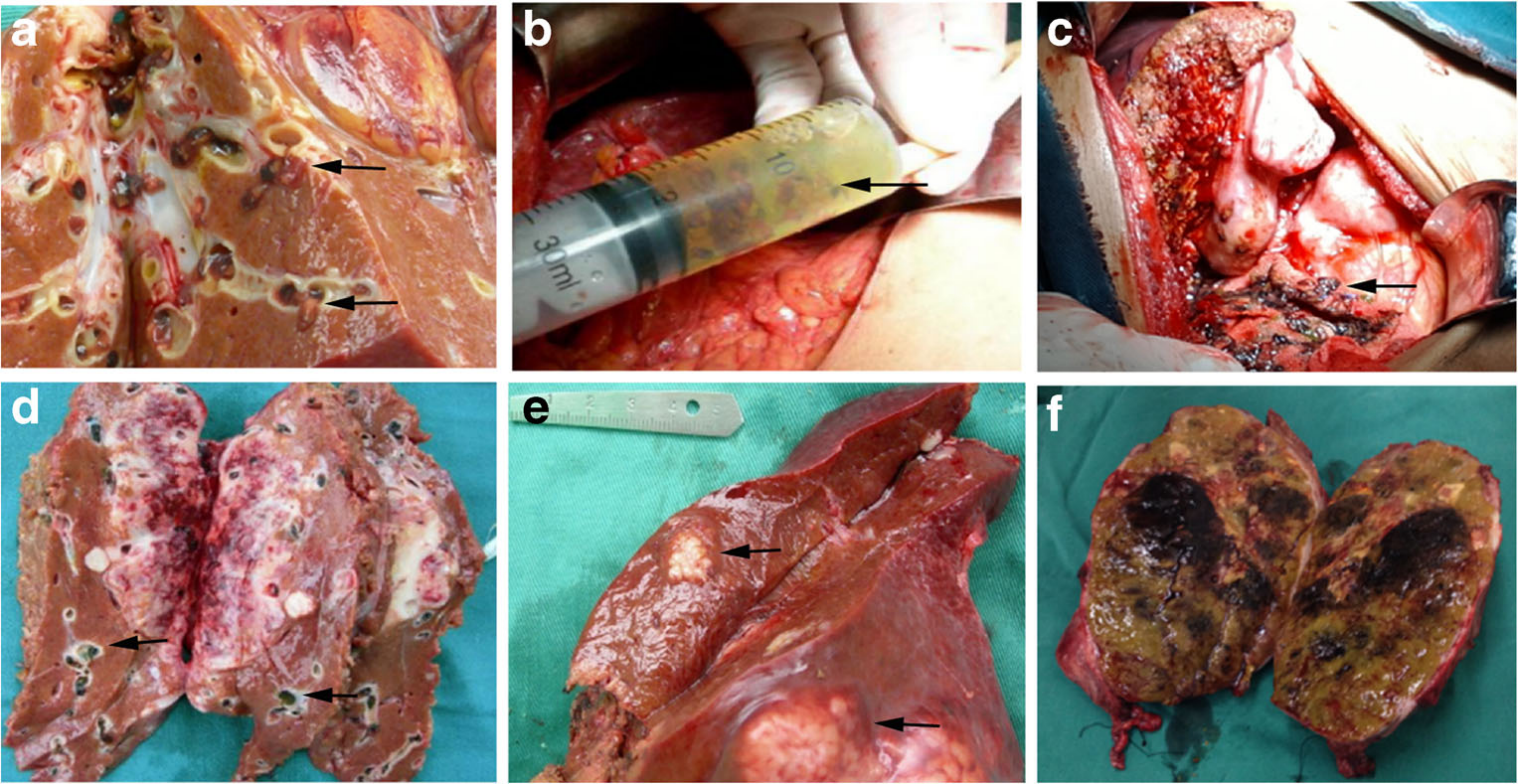
Pathologic observations of cholangiocarcinoma and hepatocellular carcinoma infected with *Clonorchis sinensis*. **a** Flukes were found in the duct during surgery. **b** Flukes were found in the bile during surgery. **c** Flukes were found in sites of liver damage. **d** Intrahepatic cholangiocarcinoma liver with *Clonorchis sinensis* infection. **e** and **f** Hepatocellular carcinoma in the liver with *Clonorchis sinensis* infection

During surgery, five patients were found to have *C*. *sinensis* in the bile duct and (or) the liver (Fig. 2a–c). Burdens of more than 200 worms, enlarged bile ducts and serious damage to the liver were found. The tumours appeared as relatively firm, grey-white masses bounded by hepatic tissues (Fig. 2e, f). Obstructed bile ducts and abnormal enhancement of the ductal wall were observed (Fig. 2d).

## Discussion

Using case investigations and retrospective data surveys, some studies reported that the incidence of ICC is significantly higher in *C*. *sinensis-*endemic areas than that in non-endemic areas (Papachristou et al. 2005; Choi et al. 2006; Lim et al. 2006), but large-scale case investigations are needed to uncover the contribution of *C*. *sinensis* to ICC and HCC. In this study, we focused on ICC and HCC in an area with a high prevalence of *C*. *sinensis* to identify the relationship between ICC and HCC and *C*. *sinensis* infection. Hengxian County is one of the most serious *C*. *sinensis*-epidemic areas located in the southern Guangxi Zhuang Autonomous Region, China. The people regularly consume raw fish even though the incidence of *C*. *sinensis* infection is high, and the infection rate has reached 53.6% (Qian et al. 2014). In this investigation, *C*. *sinensis* infection was detected in 18 of the 20 ICC and HCC patients, and only 1 patient was not infected with *C*. *sinensis* in each group, suggesting that *C*. *sinensis* is an important risk factor for ICC and HCC.

In the survey, we found that most ICC and HCC patients consumed raw fish frequently (approximately 4 times per month) and have done so for more than 10 years. Three of the female patients had not consumed raw fish before, but their families consumed raw fish frequently. Therefore, these patients were infected by contaminated food rather than the direct ingestion of raw fish. Worms were identified in five patients during surgery, with burdens of more than 50 worms and up to 200 worms in the damaged liver, indicating that the worm burden is high and may be the source of repeated infection. Carcinogenesis is a multi-step process that spans a long period of time (presumably 10–40 years) (Lee et al. 1997; Watanapa and Watanapa 2002; Kakar and Burgart 2005). A long history of repeated infection and the high intensity of *C*. *sinensis* infection may reflect important risk factors that contribute to ICC and HCC. Our study supported the classification of *C*. *sinensis* as “carcinogenic to humans” (group 1).

The mechanisms of liver fluke (*C*. *sinensis* and *Opisthorchis viverrini*) infection resulting in CCA are complicated and may include two parts (Zheng et al. 2017). The first part involves the mechanical damage caused by the activities of the parasites, generating metabolic products that can induce chronic irritation and prolonged inflammation, and infection-induced chronic inflammation, which leads to hepatobiliary or hepatic abnormalities. The second part is novel and involves microbe dysbiosis and the influence of drug-processing enzymes in the liver. Mechanisms and factors, such as granulin (Mulvenna et al. 2010) and growth factors (Sripa et al. 2012) that may be related to liver fluke-related CCA, have been proposed. However, we still need to explore the details of liver fluke involvement in the carcinogenesis of CCA and identify the differences between *C*. *sinensis* and *O*. *viverrini* in the promotion of cancer progression.

The hepatitis B virus is another, more serious pathogen that can cause liver disease and cancer. In the present study, we found that the co-infection of HBV and *C*. *sinensis* is common in *C*. *sinensis-*endemic regions. In the 20 cases of ICC and HCC, 8 were diagnosed with HBV infection and 7 were co-infected with *C*. *sinensis*. The hepatitis B prevalence is highest in East Asia, where 5–10% of the adult population is chronically infected. East Asian countries such as China, Thailand, Korea, Lao PDR and Vietnam are also high-prevalence regions for liver flukes such as *C*. *sinensis* and *O*. *viverrini*, indicating that the co-infection of liver flukes and HBV may contribute to the *C*. *sinensis* prevalence. This study showed that co-infection of HBV and *C*. *sinensis* could weaken liver function and promote HBV proliferation (Li et al. 2016). The risk factors for HBV-related HCC include host factors, viral factors and liver factors (Yang et al. 2011; Wong and Wong 2013; Wong et al. 2010). *C*. *sinensis* contributes to fibrosis, cirrhosis and poor liver function; therefore, HBV and *C*. *sinensis* co-infection will increase the risk of HCC. In addition, HBV and *C*. *sinensis* co-infection may promote HBV proliferation, and some studies have verified that an increasing HBV viral load is a strong predictor of the risk for HCC (Wong and Wong 2013; Yuen et al. 2009; Abu-Amara et al. 2016). In the present research, we also found that *C*. *sinensis* and HBV co-infection is common in ICC patients and is one of the most important factors in ICC. Whether *C*. *sinensis* and HBV co-infection has a synergistic effect on ICC remains unknown.

The potential limitations of this study include the narrow scope of the investigation and the small number of clinical cases of HCC and ICC due to limited funding and manpower resources. Additionally, in this study, we could not exclude factors that impair the liver, such as alcohol consumption, hepatitis C and hepatitis A infections; these factors may impact the liver and lead to disease and cancer. However, in the 20 cases of ICC and HCC, only one patient was not infected with *C*. *sinensis* and HBV, implying that other factors besides *C*. *sinensis* and HBV co-infection are not as closely associated with HCC and ICC in Hengxian County.

## References

[CR1] Abu-Amara M, Cerocchi O, Malhi G, Sharma S, Yim C, Shah H, Wong DK, Janssen HL, Feld JJ (2016). The applicability of hepatocellular carcinoma risk prediction scores in a North American patient population with chronic hepatitis B infection. Gut.

[CR2] Belamaric J (1973). Intrahepatic bile duct carcinoma and *C*. *sinensis* infection in Hong Kong. Cancer.

[CR3] Bouvard V, Baan R, Straif K, Grosse Y, Secretan B, El Ghissassi F, Benbrahim-Tallaa L, Guha N, Freeman C, Galichet L, Cogliano V, WHO International Agency for Research on Cancer Monograph Working Group (2009). A review of human carcinogens—part B: biological agents. Lancet Oncol.

[CR4] Chen J, Xu MJ, Zhou DH, Song HQ, Wang CR, Zhu XQ (2012). Canine and feline parasitic zoonoses in China. Parasit Vectors.

[CR5] Choi D, Lim JH, Lee KT, Lee JK, Choi SH, Heo JS, Jang KT, Lee NY, Kim S, Hong ST (2006). Cholangiocarcinoma and Clonorchis sinensis infection: a case-control study in Korea. J Hepatol.

[CR6] Honer Zu Siederdissen C, Cornberg M (2014). The role of HBsAg levels in the current management of chronic HBV infection. Ann Gastroenterol.

[CR7] Hou PC (1956). The relationship between primary carcinoma of the liver and infestation with *Clonorchis sinensis*. J Pathol Bacteriol.

[CR8] Kakar S, Burgart LJ (2005). Tumours of the biliary system. Curr Diagn Pathol.

[CR9] Lee JH, Rim HJ, Sell S (1997). Heterogeneity of the ‘oval-cell’ response in the hamster liver during cholangiocarcinogenesis following *Clonorchis sinensis* infection and dimethylnitrosamine treatment. J Hepatol.

[CR10] Li W, Dong H, Huang Y, Chen T, Kong X, Sun H, Yu X, Xu J (2016). *Clonorchis sinensis* co-infection could affect the disease state and treatment response of HBV patients. PLoS Negl Trop Dis.

[CR11] Lim MK, Ju YH, Franceschi S, Oh JK, Kong HJ, Hwang SS, Park SK, Cho SI, Sohn WM, Kim DI, Yoo KY, Hong ST, Shin HR (2006). *Clonorchis sinensis* infection and increasing risk of cholangiocarcinoma in the Republic of Korea. Am J Trop Med Hyg.

[CR12] Mulvenna J, Sripa B, Brindley PJ, Gorman J, Jones MK, Colgrave ML, Jones A, Nawaratna S, Laha T, Suttiprapa S, Smout MJ, Loukas A (2010). The secreted and surface proteomes of the adult stage of the carcinogenic human liver fluke *Opisthorchis viverrini*. Proteomics.

[CR13] Papachristou GI, Schoedel KE, Ramanathan R, Rabinovitz M (2005). Clonorchis sinensis-associated cholangiocarcinoma: a case report and review of the literature. Dig Dis Sci.

[CR14] Qian MB, Chen YD, Yang YC, Lu MF, Jiang ZH, Wei K, Wei SL, Zhou CH, Xu LQ, Zhou XN (2014). Increasing prevalence and intensity of foodborne clonorchiasis, Hengxian County, China, 1989–2011. Emerg Infect Dis.

[CR15] Qian MB, Utzinger J, Keiser J, Zhou XN (2016). Clonorchiasis. Lancet.

[CR16] Rapti I, Hadziyannis S (2015). Risk for hepatocellular carcinoma in the course of chronic *hepatitis B virus* infection and the protective effect of therapy with nucleos(t)ide analogues. World J Hepatol.

[CR17] Shin HR, Lee CU, Park HJ, Seol SY, Chung JM, Choi HC, Ahn YO, Shigemastu T (1996). *Hepatitis B* and *C virus*, *Clonorchis sinensis* for the risk of liver cancer: a case-control study in Pusan, Korea. Int J Epidemiol.

[CR18] Sripa B, Brindley PJ, Mulvenna J, Laha T, Smout MJ, Mairiang E, Bethony JM, Loukas A (2012). The tumorigenic liver fluke Opisthorchis viverrini—multiple pathways to cancer. Trends Parasitol.

[CR19] Sun Z, Ming L, Zhu X, Lu J (2002). Prevention and control of hepatitis B in China. J Med Virol.

[CR20] Tan SK, Qiu XQ, Yu HP, Zeng XY, Zhao YN, Hu L (2008). Evaluation of the risk of clonorchiasis inducing primary hepatocellular carcinoma. Zhonghua Gan Zang Bing Za Zhi.

[CR21] Watanapa P, Watanapa WB (2002). Liver fluke-associated cholangiocarcinoma. Br J Surg.

[CR22] Wong GL, Wong VW (2013). Risk prediction of hepatitis B virus-related hepatocellular carcinoma in the era of antiviral therapy. World J Gastroenterol.

[CR23] Wong VW, Chan SL, Mo F, Chan TC, Loong HH, Wong GL, Lui YY, Chan AT, Sung JJ, Yeo W, Chan HL, Mok TS (2010). Clinical scoring system to predict hepatocellular carcinoma in chronic hepatitis B carriers. J Clin Oncol.

[CR24] Yang HI, Yuen MF, Chan HL, Han KH, Chen PJ, Kim DY, Ahn SH, Chen CJ, Wong VW, Seto WK (2011). Risk estimation for hepatocellular carcinoma in chronic hepatitis B (REACH-B): development and validation of a predictive score. Lancet Oncol.

[CR25] Yang G, Han M, Chen F, Xu Y, Chen E, Wang X, Liu Y, Sun J, Hou J, Ning Q, Wang Z (2014). Hepatitis B virus genotype B and mutations in basal core promoter and pre-core/core genes associated with acute-on-chronic liver failure: a multicenter cross-sectional study in China. Hepatol Int.

[CR26] Young ND, Campbell BE, Hall RS, Jex AR, Cantacessi C, Laha T, Sohn WM, Sripa B, Loukas A, Brindley PJ, Gasser RB (2010). Unlocking the transcriptomes of two carcinogenic parasites, *Clonorchis sinensis* and *Opisthorchis viverrini*. PLoS Negl Trop Dis.

[CR27] Yuen MF, Tanaka Y, Fong DY, Fung J, Wong DK, Yuen JC, But DY, Chan AO, Wong BC, Mizokami M, Lai CL (2009). Independent risk factors and predictive score for the development of hepatocellular carcinoma in chronic hepatitis B. J Hepatol.

[CR28] Zheng S, Zhu Y, Zhao Z, Wu Z, Okanurak K, Lv Z (2017). Liver fluke infection and cholangiocarcinoma: a review. Parasitol Res.

